# Euro-Esli: a European audit of real-world use of eslicarbazepine acetate as a treatment for partial-onset seizures

**DOI:** 10.1007/s00415-017-8618-5

**Published:** 2017-09-18

**Authors:** Vicente Villanueva, Martin Holtkamp, Norman Delanty, Juan Rodriguez-Uranga, Rob McMurray, Patricia Santagueda

**Affiliations:** 1 Multidisciplinary Epilepsy Unit, Neurology Service, Hospital Universitario y Polotécnico La Fe, Avda Fernando Abril Martorell 106, 46026 Valencia, Spain; 2 Department of Neurology, Epilepsy-Center Berlin-Brandenburg, Charité–Universitätsmedizin, Charitéplatz 1, 10117 Berlin, Germany; 3 Division of Neurology, Beaumont Hospital, Beaumont Road, Dublin 9, Ireland; 4 Centro de Neurología Avanzada, Avenida Manuel Siurot no 43-A, 41013 Seville, Spain; 5 European Knowledge Centre, Eisai Europe Ltd, Mosquito Way, Hatfield, Hertfordshire AL10 9SN UK; 6 Refractory Epilepsy Unit, Hospital Universitario y Politécnico La Fe, 46026 Valencia, Spain

**Keywords:** Antiepileptic drug, Clinical practice, Epilepsy, Eslicarbazepine acetate, Pooled analysis, Real world

## Abstract

The Euro-Esli study was an exploratory pooled analysis of data from 14 European clinical practice studies, which was conducted to audit the real-world effectiveness, safety, and tolerability of eslicarbazepine acetate (ESL) as an adjunctive treatment for partial-onset seizures. Retention and effectiveness were assessed after 3, 6, and 12 months of ESL treatment, and at the final visit. Safety and tolerability were assessed throughout ESL treatment by evaluating adverse events (AEs) and ESL discontinuation due to AEs. Data from 2058 patients (52.1% male; mean age 44.0 years) were included. All 2058 patients were assessed for safety and 1975 (96.0%) patients were assessed for effectiveness. After 12 months, retention, responder (≥50% seizure frequency reduction), and seizure freedom rates were 73.4, 75.6, and 41.3%, respectively. AEs were reported for 34.0% of patients and led to discontinuation in 13.6% of patients. The most frequently reported AEs were dizziness (6.7% of patients), fatigue (5.4%), and somnolence (5.1%). No unexpected safety signals emerged over a median duration of follow-up of >5 years. Subgroup analyses revealed that ESL was significantly more effective in patients aged ≥65 versus <65 years, in patients who were not receiving treatment with other sodium channel blockers versus those who were receiving treatment with other sodium channel blockers, and in patients who were receiving <2 versus ≥2 concomitant antiepileptic drugs at baseline. Euro-Esli is the largest ESL clinical practice study conducted to date. This study provides strong and reassuring evidence of ESL’s safety profile, and complements the data from clinical trials.

## Introduction

Eslicarbazepine acetate (ESL) is a once-daily antiepileptic drug (AED) that is approved for the treatment of partial-onset seizures as monotherapy or adjunctive therapy [1, 2]. It is thought to act primarily by enhancing slow inactivation of voltage-gated sodium channels [3]. The efficacy of ESL as adjunctive therapy for partial-onset seizures in adults, together with its safety and tolerability profile in this setting, was established in a series of randomized, double-blind, placebo-controlled, Phase III trials [4–7] and long-term extension studies [8–10]. The efficacy, safety, and tolerability of ESL as monotherapy for the treatment of partial-onset seizures in adults with newly diagnosed epilepsy were established in a Phase III, randomized, double-blind, active-controlled, non-inferiority trial [11, 12]. In addition, the efficacy, safety, and tolerability of ESL as monotherapy for adults with uncontrolled partial-onset seizures were established in two randomized, Phase III, withdrawal to monotherapy trials [13, 14].

Although clinical trials are essential in the development and approval of new AEDs, they do not necessarily reflect the effectiveness and tolerability of an agent when used in clinical practice. This is due to clinical trials selecting relatively homogeneous patient populations and typically using rigid dosing and titration schedules, whereas patients encountered in clinical practice have diverse clinical characteristics that necessitate an individualized approach to treatment [15, 16]. Consequently, there is a need for real-world studies to complement evidence from clinical trials, by elucidating an agent’s effectiveness when used under everyday clinical practice conditions.

The aim of the Euro-Esli study was to conduct an audit of data from clinical practice studies conducted across Europe to establish how the efficacy and tolerability of ESL observed in clinical trials have translated into effectiveness in the real-world setting. The study involved the collaboration of a pan-European group of clinicians and centers, resulting in the largest database of patients treated with ESL in everyday clinical practice to date. The pooling of a large body of data also enabled subgroup analyses to be conducted, which allowed specific aspects of the use of ESL in epilepsy management to be addressed. We present here the primary results of the audit, together with those of some key subgroup analyses.

## Methods

### Study design

The Euro-Esli study was an exploratory, pooled analysis of data from European clinical practice studies (both prospective and retrospective) that evaluated the effectiveness, safety, and tolerability of ESL as an adjunctive treatment for partial-onset seizures. There were no exclusion criteria for the studies chosen for the analysis in terms of the epilepsy type of the patients studied and/or the number of prior AEDs they had received. Effectiveness was assessed after 3, 6, and 12 months of ESL treatment and at final follow-up. Safety and tolerability were assessed for the duration of ESL treatment. The study protocol was approved by the Ethics Committee of the Hospital Universitario y Politécnico La Fe, Valencia, Spain, as an extension of the local audit.

### Study population

Details of the specific inclusion/exclusion criteria used in the individual studies have been published or presented previously [17–29]. The studies included broad inclusion/exclusion criteria, to be representative of the variety of patients encountered in clinical practice.

The current analysis included all patients who initiated ESL for the treatment of epilepsy. Most patients were treated for partial-onset seizures, although patients with generalized seizures were not specifically excluded. However, analyses of effectiveness focussed on partial-onset seizures, with or without secondary generalization. Patients were excluded if their records contained insufficient data for analysis. Duplicate data from patients who were included in more than one study were excluded.

### Study assessments

#### Effectiveness

Assessments of effectiveness comprised: retention, evaluated in terms of retention rate and time to ESL discontinuation; number and type of seizures (total, simple partial, complex partial, and secondarily generalized seizures [30]); the percentage reduction from baseline in monthly seizure frequency (for total, simple partial, complex partial, and secondarily generalized seizures); responder rate; seizure freedom rate; and the percentage of patients whose seizure frequency remained unchanged and whose seizure frequency worsened, relative to baseline. At baseline (i.e., prior to ESL initiation), monthly seizure frequency was calculated based on the number of seizures experienced during the previous 3 months. At other timepoints, monthly seizure frequency was based on the number of seizures experienced since the previous visit, i.e., during the previous 3 months for the 3- and 6-month visits and during the previous 6 months for the 12-month visit. For the final assessment, monthly seizure frequency was based on the last visit, which could have been at 3, 6, or 12 months; therefore, seizure frequency at the last visit was based on the number of seizures experienced during at least the previous 3 months. Response was defined as ≥50% seizure frequency reduction from baseline and seizure freedom was defined as the occurrence of no seizures since at least the prior visit (either 3 or 6 months).

#### Safety and tolerability

Safety was assessed by the evaluation of adverse events (AEs) and tolerability was assessed by evaluating the discontinuation of ESL due to AEs. AEs reported by participating clinicians were also classified using the Medical Dictionary for Regulatory Activities version 16.0 [31]. AEs were classified as being related to ESL treatment if they started after ESL initiation and were considered by the participating clinician(s) to be ESL-related. Certain AEs of special interest were also assessed, which were hyponatremia, psychiatric AEs, and the presence of ESL-related AEs in patients with poor tolerance to previous AEDs.

#### Additional assessments

Additional assessments included evaluation of information relating to ESL dosing, ESL treatment adherence, and changes to concomitant treatment(s) (pharmacological and non-pharmacological) after initiation of ESL treatment. Information on treatment adherence was collected in clinical charts.

### Subgroup analyses

Several subgroup analyses of the pooled data were conducted. These included, first, analysis of data for elderly patients (≥65 years) versus non-elderly patients (<65 years); second, comparison of the subgroups of patients who were using, versus not using, other sodium channel blockers (SCBs) during ESL treatment; and third, assessment of outcomes in terms of how refractory to treatment the patients were at baseline, where refractoriness was defined in terms of the number of baseline AEDs patients were receiving when ESL was initiated (less than two versus at least two concomitant AEDs).

For all subgroup analyses, effectiveness was assessed as responder and seizure freedom rates (as previously defined), safety was assessed by evaluating AEs, and tolerability was assessed by evaluating discontinuation of ESL due to AEs.

### Statistical analyses

The safety population was defined as all patients who initiated ESL treatment. The efficacy population was defined as all patients who initiated ESL treatment and had at least one efficacy assessment.

There was great heterogeneity in the particular objectives of the studies included in the analysis and, thus, in the information each study reported. The current analysis attempted to combine the reported information in the most complete way possible. Missing data were not imputed, except in cross-sectional studies, in which the last visit data were captured and included in the established cut-off points (3, 6, or 12 months). When the observation timepoint of a study did not match the established cut-off points, the following allocations were made: observations performed between 1.5 and <4.5 months were allocated to the 3-month visit; those performed between 4.5 and <9 months were allocated to the 6-month visit; and those performed between 9 and 15 months were allocated to the 12-month visit. A ‘final’ variable was also created, in which the last observation of each patient was included, independently of the timepoint when it occurred. Since this was an exploratory study, no hypothesis was defined. No systematic review of the individual patients was undertaken due to the heterogeneity of the individual samples and objectives of each study. Therefore, individual studies were not treated as clusters. Similarly, adjustments at the significance level were not considered due to multiple comparisons.

A descriptive analysis of quantitative and qualitative variables was performed. For each variable, the total number of patients for whom the data in question were available was stated and this value was used as the denominator for analysis. Quantitative variables were described as mean, standard deviation (SD), median, minimum and maximum values, together with the number of valid cases and confidence intervals (CIs) or interquartile range (25th percentile to 75th percentile). Qualitative variables were described as means of absolute frequencies and percentages. Variation in the number of seizures/month between baseline and the final timepoint was assessed using the Wilcoxon signed-rank test and variation in the type of seizures was assessed using McNemar’s test. Treatment response and safety and tolerability assessments were studied as a function of the different subpopulations using the Chi-squared test or Fisher’s exact test, as appropriate. Time to ESL discontinuation was assessed using the Kaplan–Meier method. ESL dose variation between baseline and the final timepoint was assessed using the Student’s *t* test for repeated measures. Variation between the initial and final number of concomitant AEDs was assessed using the Wilcoxon signed-rank test. The Statistical Package for the Social Sciences version 19.0 was used for all analyses. The significance level was 5%.

## Results

A total of 14 European clinical practice studies were included in the analysis, details of which are presented in Table 1. Information was gathered from 2079 patients with epilepsy who had initiated treatment with ESL. A total of 20 patients were excluded due to insufficient data. Duplicate data from one patient who was included in two studies were also excluded. The final sample, therefore, included 2058 patients.

**Table 1:** Overview of studies included in the pooled analysis

Name	Country/countries	Design	Effectiveness assessments	Number of patients included in analysis/total number of patients
Barcelona audit [17, 18]	Spain	Cross-sectional	Seizure frequency	109/109
Coimbra [19]	Portugal	Longitudinal/retrospective	Seizure frequency	122/140
Early-Esli [20]	Spain	Longitudinal/retrospective	SF, *R* ≥50%, *R* <50%, stay the same, worse	252/253
EPOS [21]	Czech Republic, Denmark, France, Germany, Ireland, Norway, Sweden, United Kingdom	Longitudinal/prospective	Seizure frequency	245/247
ESLADOBA [22]	Portugal	Longitudinal/prospective	Seizure frequency	52/52
ESLIBASE [23]	Spain	Longitudinal/retrospective	SF, *R* ≥50%, *R* <50%, stay the same, worse	327/327
German audit [24]	Germany	Longitudinal/retrospective	Seizure frequency	125/125
Ireland audit [25]	Ireland	Cross-sectional	Seizure frequency	217/217
Italy audit [26]	Italy	Cross-sectional	Mean seizure frequency	69/69
Lyon audit [unpublished^a^, 2015]	France	Longitudinal/retrospective	SF, *R* ≥50%	64/64
NEON [unpublished^b^, 2014]	Czech Republic	Longitudinal/retrospective	Seizure frequency	201/201
ROME [27]	Italy	Cross-sectional	SF, *R* ≥50%, *R* <50%, stay the same, worse	50/50
Tampere audit [28]	Finland	Longitudinal/retrospective	Responder/non-responder	23/23
UK audit [29]	United Kingdom	Longitudinal/retrospective	Seizure frequency	202/202
				Total: 2058/2079

^a^ Rheims S. A retrospective evaluation of ESL in France. Presented at the Annual Meeting of the French ILAE Chapter, Montpellier, France, 2015

^b^ Study was not published or presented, since it was used for local reimbursement in the Czech Republic

*EPOS* eslicarbazepine acetate in partial-onset seizures, *ESL* eslicarbazepine acetate, *ILAE* International League Against Epilepsy, *NEON* an observational, long-term, multicentre, post-marketing, non-interventional study of the use of eslicarbazepine acetate in the adjunctive treatment of adult patients with partial-onset seizures, *ROME* retrospective observational multicentre study on ESL, *R* ≥50%, responder rate (response defined as ≥50% seizure frequency reduction); *R* <50%, responder rate (response defined as <50% seizure frequency reduction), *SF* seizure freedom rate

### Patient disposition

**Fig. 1:**
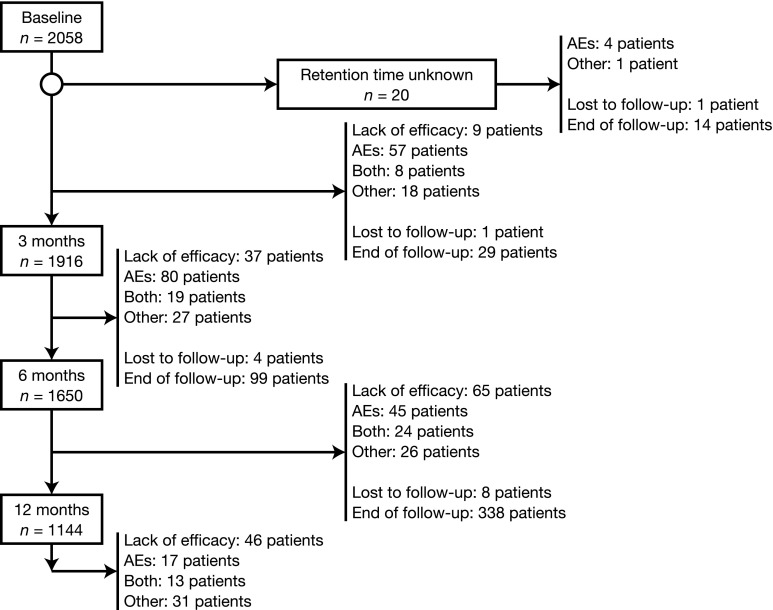
Patient disposition. *AE* adverse event

Of the 2058 patients included in the analysis, 1916 (93.1%), 1650 (80.2%), and 1144 (55.6%) received 3, 6, and 12 months of ESL treatment, respectively, and information on retention time was unknown for 20 (1.0%) patients (Fig. 1). The safety population included all 2058 patients. The efficacy population included 1975 (96.0%) patients who had at least one efficacy assessment.

### Study population

The mean age of the study population was 44.0 years and 52.1% of patients were male (Table 2). The mean age at onset of epilepsy was 23.3 years and the mean duration of epilepsy was 20.9 years. Patients had been treated with a median of 3.0 other AEDs prior to starting ESL therapy; most commonly (≥40% of patients), levetiracetam (64.3%), lamotrigine (45.3%), carbamazepine (45.2%), and valproate (41.9%). At the time of ESL initiation, most patients were receiving one (47.4%) or two (27.7%) concomitant AEDs. The most frequently used concomitant AEDs (≥20% of patients) were levetiracetam (40.3%) and lamotrigine (23.5%). Of the patients for whom the mode of action of concomitant AEDs was known, 53.6% were being treated with another SCB at the time of ESL initiation.

**Table 2:** Patient demographics and baseline characteristics

Baseline demographics	
Sex	
*N* ^a^	2057
Male, *n* (%)	1071 (52.1)
Female, *n* (%)	986 (47.9)
Age	
*N* ^a^	2057
Mean (SD), years	44.0 (15.5)
Median (range), years	42.4 (14–88)
Age category	
<18 years, *n* (%)	12 (0.6)
18–64 years, *n* (%)	1804 (87.7)
≥65 years, *n* (%)	241 (11.7)
Epilepsy-related characteristics	
Age at onset of epilepsy	
*N* ^a^	1861
Mean (SD), years	23.3 (19.2)
Median (range), years	18.0 (0.0–87.0)
Duration of epilepsy	
*N* ^a^	1861
Mean (SD), years	20.9 (16.4)
Median (range), years	18.0 (0.0–81.8)
Etiology (ILAE 2010 classification)	
*N* ^a^	1656
Structural–metabolic	947 (57.2)
Genetic	36 (2.2)
Unknown	673 (40.6)
Baseline seizure type	
Any seizure	
*N* ^a^	1991
Yes, *n* (%)	1853 (93.1)
Simple partial seizures	
*N* ^a^	1834
Yes, *n* (%)	478 (26.1)
Complex partial seizures	
*N* ^a^	1834
Yes, *n* (%)	1137 (62.0)
Secondarily generalized seizures	
*N* ^a^	1834
Yes, *n* (%)	785 (42.8)
Baseline monthly seizure frequency	
Any seizure	
*N* ^a^	1853
Mean (SD)	13.6 (49.9)
Median (range)	3.0 (0.1–1230.0)
Simple partial seizures	
*N* ^a^	396
Mean (SD)	14.6 (59.7)
Median (range)	3.0 (0.3–900.0)
Complex partial seizures	
*N* ^a^	890
Mean (SD)	8.3 (22.1)
Median (range)	2.8 (0.2–300.0)
Secondarily generalized seizures	
*N* ^a^	626
Mean (SD)	2.5 (6.2)
Median (range)	0.9 (0.1–70.0)
Comorbidities	
Intellectual disability	
*N* ^a^	952
Yes, *n* (%)	108 (11.3)
Psychiatric comorbidity (including depression)	
*N* ^a^	1138
Yes, *n* (%)	283 (24.9)
Depression	
*N* ^a^	1134
Yes, *n* (%)	141 (12.4)
AED treatment	
Total number of previous AEDs	
*N* ^a^	1897
Mean (SD)	4.1 (3.4)
Median (range)	3.0 (0–20)
Number of previous AEDs	
*N* ^a^	1897
0, *n* (%)	20 (1.1)
1, *n* (%)	474 (25.0)
2, *n* (%)	336 (17.7)
3, *n* (%)	246 (13.0)
4, *n* (%)	162 (8.5)
5, *n* (%)	167 (8.8)
>5, *n* (%)	492 (25.9)
Most frequently used (≥20% patients) previous AEDs	
*N* ^a^	1200
Levetiracetam, *n* (%)	772 (64.3)
Lamotrigine, *n* (%)	543 (45.3)
Carbamazepine, *n* (%)	542 (45.2)
Valproate, *n* (%)	503 (41.9)
Clobazam, *n* (%)	338 (28.2)
Zonisamide, *n* (%)	274 (22.8)
Lacosamide, *n* (%)	262 (21.8)
Topiramate, *n* (%)	262 (21.8)
Total number of concomitant AEDs	
*N* ^a^	2045
Mean (SD)	1.7 (1.0)
Median (range)	1.0 (0–6)
Number of concomitant AEDs	
*N* ^a^	2045
0, *n* (%)	88 (4.3)
1, *n* (%)	969 (47.4)
2, *n* (%)	567 (27.7)
3, *n* (%)	281 (13.7)
4, *n* (%)	112 (5.5)
5, *n* (%)	24 (1.2)
6, *n* (%)	4 (0.2)
Most frequently used (≥5% patients) concomitant AEDs	
*N* ^a^	1940
Levetiracetam, *n* (%)	782 (40.3)
Lamotrigine, *n* (%)	455 (23.5)
Valproate, *n* (%)	376 (19.4)
Carbamazepine, *n* (%)	289 (14.9)
Clobazam, *n* (%)	275 (14.2)
Lacosamide, *n* (%)	219 (11.3)
Zonisamide, *n* (%)	193 (9.9)
Topiramate, *n* (%)	140 (7.2)
Oxcarbazepine, *n* (%)	102 (5.3)
Use of concomitant SCB(s) at baseline	
*N* ^a^	1852
Yes, *n* (%)	993 (53.6)

^a^ *N* refers to the total number of patients for whom data in question were available

*AED* antiepileptic drug, *ILAE* International League Against Epilepsy, *SCB* sodium channel blocker, *SD* standard deviation

### ESL dosing

ESL was initiated for the treatment of partial-onset seizures in all except three patients. The most frequent reason for initiating ESL was lack of effectiveness of previous treatment (73.9%; 983/1330), followed by poor tolerance to previous treatment (14.1%; 187/1330). In addition, 8.4% (112/1330) of patients initiated ESL due to lack of effectiveness plus poor tolerance to previous treatment, and 3.6% (48/1330) of patients initiated ESL due to other reasons [most commonly, lack of compliance with previous treatment (*n* = 27)].

The mean (SD) baseline ESL dose was 529.2 (248.6) mg/day (median 400; range 150–1600). The maximum mean (SD) ESL dose reached was 987.0 (326.4) mg/day (median 800; range 300–2800). At the last visit, the mean (SD) ESL dose was 978.2 (328.9) mg/day (median 800; range 200–2800). There was a significant increase in the mean ESL dose used from baseline to the last visit (*p* < 0.001; *t* = 34.43; Student’s *t* test for repeated measures). The median ESL dose was 400 mg/day at baseline and 800 mg/day from month 3 onwards. A total of 171/1920 (8.9%) and 18/1920 (0.9%) patients received an ESL dose of >1200 and >1600 mg/day at some point during the observation period, respectively. Information regarding adherence to treatment was available for 300 patients. Good adherence to treatment (as judged by the participating clinicians) was reported for 95.7% (287/300) of patients.

There was a significant reduction in the number of concomitant AEDs used from baseline to the last visit (*p* < 0.001; |*Z*| = 17.61; Wilcoxon signed-rank test). The mean (SD) number of concomitant AEDs was 1.7 (1.0) at baseline (median 1.0; range 0–6; *n* = 2045) and 1.4 (1.0) at the last visit (median 1.0; range 0–6; *n* = 1340). Overall, 4.3% (88/2045) of patients received ESL as initial monotherapy at baseline. At the last visit, 17.1% (229/1340) of patients were receiving ESL as monotherapy.

### Effectiveness

#### Retention rate

The median duration of ESL treatment was 65 months (95% CI 56.7–73.3; Kaplan–Meier analysis) and the mean duration was 50.3 months (95% CI 45.2–55.5; Kaplan–Meier analysis). Overall, 26.1% (527/2018) of patients discontinued ESL during the observation period. Reasons for ESL discontinuation were AEs (10.1%; *n* = 203), lack of efficacy (7.8%; *n* = 157), AEs and lack of efficacy (3.2%; *n* = 64), other [2.2%; *n* = 45; most commonly, at the request of the patient (*n* = 9), due to lack of compliance (*n* = 3), and due to cost (*n* = 3)], and unknown (2.9%; *n* = 58).

Retention on ESL treatment at 3, 6, and 12 months was 95.4% (1916/2008), 86.6% (1650/1905), and 73.4% (1144/1559), respectively. Since most patients were not followed after 12 months (whether or not they continued ESL treatment), retention at 24 and 36 months was calculated only using the studies with >12 months of follow-up data. In these studies, retention rates at 24 and 36 months were 43.4% (228/525) and 29.8% (107/359), respectively. In the cohort of patients with 36 months of follow-up data, reasons for ESL discontinuation were lack of efficacy (20.6%; *n* = 74), AEs (22.6%; *n* = 81), AEs and lack of efficacy (8.1%; *n* = 29), other (5.3%; *n* = 19), and unknown (13.6%; *n* = 49). Overall, 73.9% (1491/2018) of patients were retained on ESL treatment when the last observation was performed.

#### Percentage of patients with seizures: total seizures and by seizure type

**Fig. 2:**
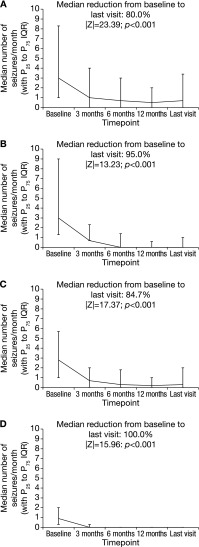
Median monthly seizure frequencies (with P_25_ and P_75_) at baseline, 3, 6, 12 months, and the last visit for **a** total seizures, **b** simple partial seizures, **c** complex partial seizures, and **d** secondarily generalized seizures. *IQR* interquartile range; P_25_, 25th percentile; P_75_, 75% percentile

At the time of ESL initiation, a total of 93.1% (1853/1991) of patients presented having had at least one seizure in the past 3 months. The monthly total seizure frequency decreased significantly from a median of 3.0 [mean (SD), 13.6 (49.9); range 0.1–1230.0] at baseline to 0.7 [mean (SD), 7.6 (34.4); range 0.0–3.4] at the last visit (|*Z*| = 23.39; *p* < 0.001; Wilcoxon signed-rank test) (Fig. 2a). The mean reduction from baseline to the last visit was 44.1% (median 80.0%).

The percentage of patients with simple partial seizures [i.e., the percentage of patients who experienced at least one simple partial seizure during an assessment period of ≥3 months (see “Methods”)] decreased significantly from 26.1% (478/1834) at baseline to 16.9% (228/1353) at the last visit (*p* < 0.001; McNemar’s test). Similarly, the percentage of patients with complex partial seizures decreased from 62.0% (1137/1834) to 36.5% (494/1353; *p* < 0.001), and the percentage of patients with secondarily generalized seizures decreased from 42.8% (785/1834) to 11.1% (150/1353; *p* < 0.001). There were significant reductions from baseline to last visit in the monthly frequencies of simple partial, complex partial, and secondarily generalized seizures [mean (median) reductions, 78.8% (95.0%), 53.1% (84.7%), and 80.0% (100.0%), respectively; *p* < 0.001 for all; Wilcoxon signed-rank test] (Fig. 2b–d).

#### Responder and seizure freedom rates

**Fig. 3:**
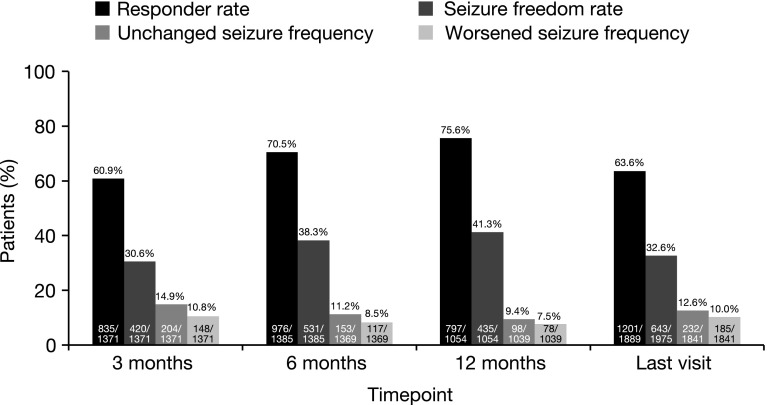
Responder rate, seizure freedom rate, and percentage of patients with unchanged or worsened seizure frequency (relative to baseline) at 3, 6, 12 months, and the last visit. Response was defined as ≥50% seizure frequency reduction from baseline. Seizure freedom was defined as no seizures since at least the prior visit; therefore, seizure freedom rates at 3 months, 6 months, and the last visit represent the percentages of patients who had no seizures for ≥3 months, and the seizure freedom rate at 12 months represents the percentage of patients who had no seizures for ≥6 months

The responder rate was 75.6% at 12 months and 63.6% at the last visit (Fig. 3). The seizure freedom rate was 41.3% at 12 months and 32.6% at the last visit (Fig. 3). A total of 217 patients presented with no seizures during follow-up (although in some cases only the last visit was recorded). The duration of observation for these patients ranged from 6.5 to 66 months. Of these 217 patients, 49 (22.6%) did not present with seizures at baseline.

#### Percentage of patients with unchanged or worsened seizure frequency

The percentages of patients with unchanged or worsened seizure frequency, relative to baseline, remained generally stable at all timepoints (Fig. 3). The percentage of patients with unchanged seizure frequency was 9.4% at 12 months and 12.6% at the last visit. The percentage of patients with worsened seizure frequency was 7.5% at 12 months and 10.0% at the last visit.

### Safety and tolerability

#### AEs and discontinuation of ESL due to AEs

Overall, 34.0% (691/2031) of patients reported AEs at some point during follow-up (Table 3). The type of AE was known in 622/691 (90.0%) patients. In terms of system organ classes, the most frequently reported AEs (≥3% of patients) were nervous system disorders (17.9%), followed by general disorders and administration site conditions (5.9%), metabolism and nutrition disorders (4.1%), psychiatric disorders (3.3%), and gastrointestinal disorders (3.1%). The most frequently reported individual AEs (≥3% of patients) were dizziness (6.7%), fatigue (5.4%), somnolence (5.1%), hyponatremia (3.5%), instability/ataxia (3.4%), and diplopia/blurred vision (3.0%).

**Table 3:** Summary of AEs

Patients with AEs	
*N* ^a^	2031
*n* (%)	691 (34.0)
Most frequently reported AEs (≥1% patients)	
*N* ^a^	1962
Dizziness, *n* (%)	132 (6.7)
Fatigue, *n* (%)	105 (5.4)
Somnolence, *n* (%)	100 (5.1)
Hyponatremia, *n* (%)	68 (3.5)
Instability/ataxia, *n* (%)	67 (3.4)
Diplopia/blurred vision, *n* (%)	58 (3.0)
Rash, *n* (%)	44 (2.2)
Nausea, *n* (%)	37 (1.9)
Disturbance in attention/concentration, *n* (%)	36 (1.8)
Headache, *n* (%)	35 (1.8)
Gait disturbance, *n* (%)	20 (1.0)
Tremor, *n* (%)	20 (1.0)
Patients with AEs leading to ESL discontinuation	
*N* ^a^	1960
*n* (%)	267 (13.6)
Most frequently reported AEs leading to ESL discontinuation (≥1% patients)	
*N* ^a^	1962
Dizziness, *n* (%)	46 (2.3)
Fatigue, *n* (%)	39 (2.0)
Rash, *n* (%)	30 (1.5)
Somnolence, *n* (%)	29 (1.5)
Instability/ataxia, *n* (%)	22 (1.1)
Diplopia/blurred vision, *n* (%)	22 (1.1)
Nausea, *n* (%)	21 (1.1)
Disturbance in attention/concentration, *n* (%)	20 (1.0)
Hyponatremia, *n* (%)	19 (1.0)

^a^ *N* refers to the total number of patients for whom data in question were available

*AE* adverse event, *ESL* eslicarbazepine acetate

AEs led to discontinuation of ESL in 13.6% (267/1960) of patients. The type of AE leading to ESL discontinuation was known in 265 patients. In terms of system organ classes, the AEs most frequently leading to ESL discontinuation (≥1% of patients) were nervous system disorders (5.9%), followed by general disorders and administration site conditions (2.3%), skin and subcutaneous tissue disorders (1.7%), gastrointestinal disorders (1.5%), psychiatric disorders (1.3%), eye disorders (1.1%), and metabolism and nutrition disorders (1.1%). The individual AEs that most frequently led to ESL discontinuation (≥1% of patients) were dizziness (2.3%), fatigue (2.0%), rash (1.5%), somnolence (1.5%), instability/ataxia (1.1%), diplopia/blurred vision (1.1%), nausea (1.1%), disturbance in attention/concentration (1.0%), and hyponatremia (1.0%).

#### AEs of special interest

Hyponatremia was reported as an AE for 68/1962 (3.5%) patients. Among the 52 of these patients who had sodium levels recorded, the mean (SD) sodium level was 127.3 (4.5) mEq/L (95% CI, 126.0–128.6; median, 127.0; range, 117–137). Thirty-four patients had sodium levels < 130 mEq/L, including three patients with sodium levels ≤120 mEq/L (117 mEq/L, *n* = 1; 118 mEq/L, *n* = 1; 120 mEq/L, *n* = 1).

Psychiatric AEs were reported for 64/1962 (3.3%) patients. Of these 64 patients, the presence/absence of psychiatric comorbidity was recorded in 25 patients, 10 (40.0%) of whom presented with previous psychiatric disease. Of the 1898 patients who did not experience a psychiatric AE, the presence/absence of psychiatric comorbidity was known in 1069 patients, 262 (24.5%) of whom presented with previous psychiatric disease. No association between the occurrence of psychiatric AEs and the presence of previous psychiatric disease was found (*χ* ^2^ = 3.14; *p* = 0.076; Chi-squared test).

A total of 299 patients initiated ESL due to poor tolerance to previous AEDs, in 276 of whom the presence or absence of ESL-related AEs was recorded, and in 291 of whom the presence or absence of ESL discontinuation due to AEs was recorded. ESL-related AEs were reported for 99/276 (35.9%) patients. ESL withdrawal due to AEs occurred in 35/291 (12.0%) patients.

### Subgroup analyses

#### Elderly patients

**Fig. 4:**
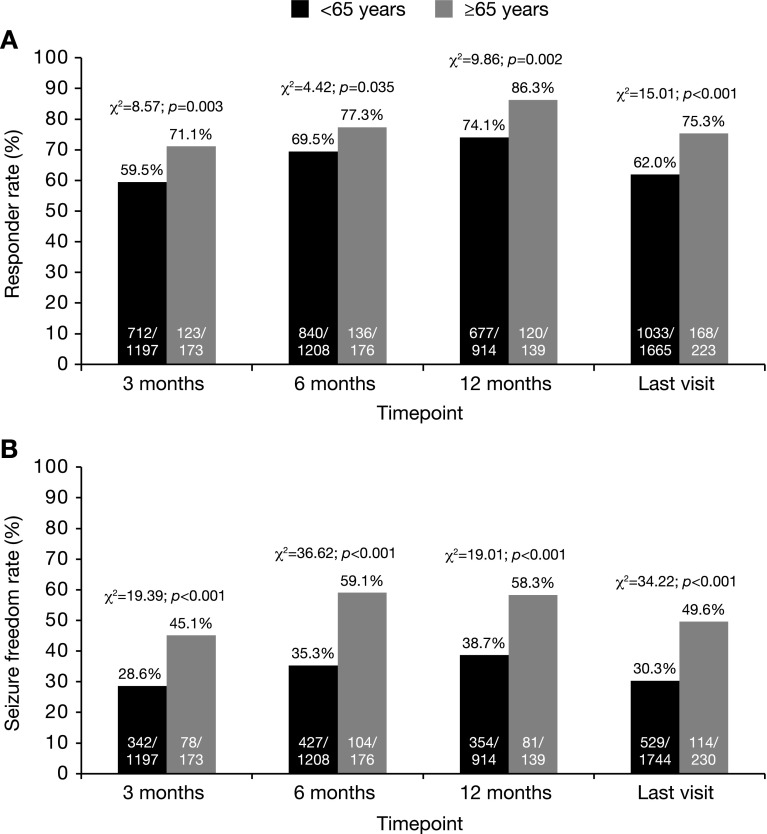
Effectiveness in elderly (≥65 years) versus non-elderly (<65 years) patients: **a** responder rate and **b** seizure freedom rate at 3, 6, 12 months, and the last visit. Response was defined as ≥50% seizure frequency reduction from baseline. Seizure freedom was defined as no seizures since at least the prior visit; therefore, seizure freedom rates at 3, 6 months, and the last visit represent the percentages of patients who had no seizures for ≥3 months, and the seizure freedom rate at 12 months represents the percentage of patients who had no seizures for ≥6 months. Statistical comparisons were conducted using the Chi-squared test

Overall, 11.7% (241/2057) of patients were aged ≥65 years and 88.3% (1816/2057) were aged <65 years. At all timepoints, responder and seizure freedom rates were significantly greater in elderly patients (≥65 years) compared with patients aged <65 years (Fig. 4).

The incidence of AEs was significantly greater in patients aged ≥65 years (43.9%; 105/239) compared with those aged <65 years (32.7%; 586/1791; *χ* ^2^ = 11.81; *p* = 0.001; Chi-squared test). The rate of ESL discontinuation due to AEs in patients aged ≥65 years (16.4%; 37/225) was similar to that of patients aged <65 years (13.3%; 230/1734; *χ* ^2^ = 1.71; *p* = 0.191; Chi-squared test).

#### Patients using SCBs versus not using other SCBs

**Fig. 5:**
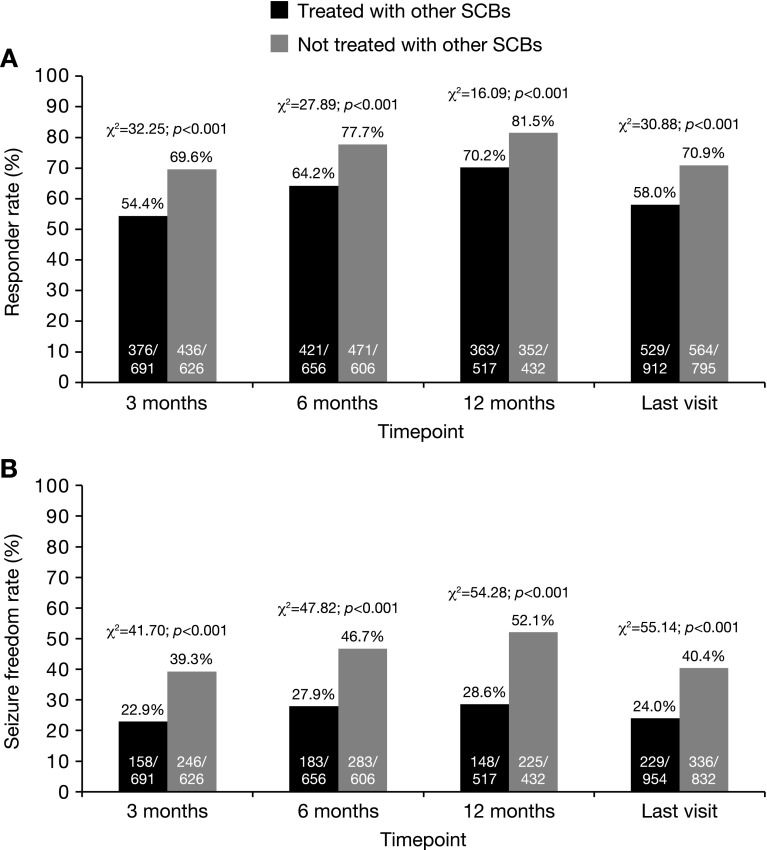
Effectiveness in patients receiving SCBs versus not receiving other SCBs: **a** responder rate and **b** seizure freedom rate at 3, 6, 12 months, and the last visit. Response was defined as ≥50% seizure frequency reduction from baseline. Seizure freedom was defined as no seizures since at least the prior visit; therefore, seizure freedom rates at 3, 6 months, and the last visit represent the percentages of patients who had no seizures for ≥3 months, and the seizure freedom rate at 12 months represents the percentage of patients who had no seizures for ≥6 months. Statistical comparisons were conducted using the Chi-squared test. *SCB* sodium channel blocker

The pooled analysis included 1852 patients for whom the mode of action of concomitant AED treatment was known, and 993 (53.6%) of these patients were treated with SCBs when ESL was initiated (Table 2). At all timepoints, responder and seizure freedom rates were significantly greater in patients who were not receiving treatment with other SCBs, compared with those who were receiving treatment with other SCBs (Fig. 5).

The overall incidence of AEs was similar among patients treated with other SCBs (35.3%; 349/989) and those not treated with other SCBs (33.1%; 284/859; *χ* ^2^ = 1.01; *p* = 0.314; Chi-squared test). However, the rate of ESL discontinuation due to AEs was significantly higher in patients treated with other SCBs (16.5%; 157/953) compared with those not treated with other SCBs (12.2%; 100/823; *χ* ^2^ = 6.67; *p* = 0.010; Chi-squared test).

#### Treatment refractoriness

**Fig. 6:**
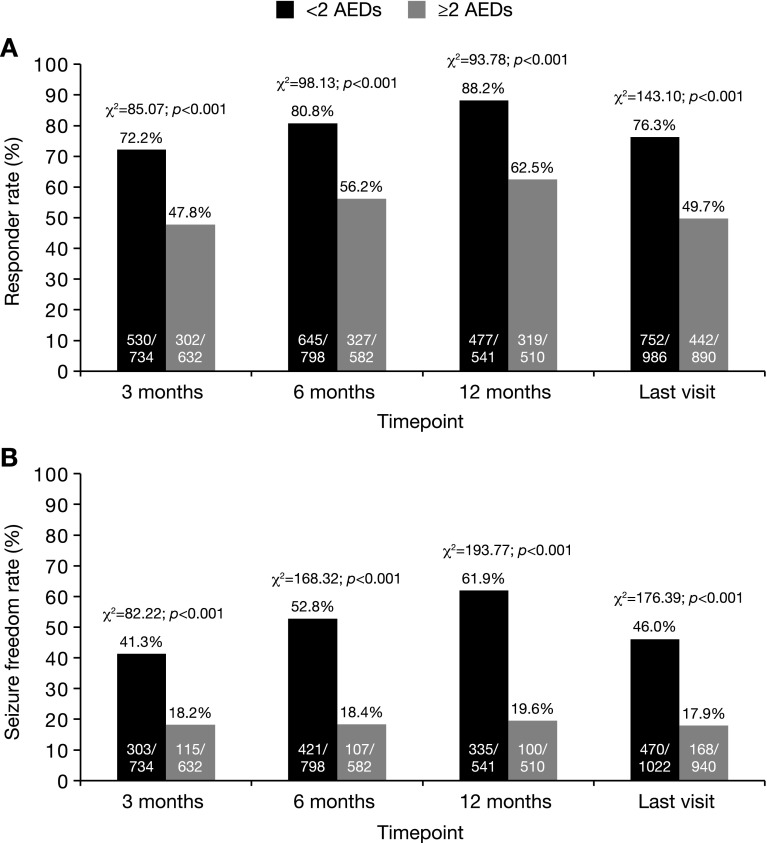
Effectiveness in patients receiving less than two versus at least two concomitant AEDs at baseline: **a** responder rate and **b** seizure freedom rate at 3, 6, 12 months, and the last visit. Response was defined as ≥50% seizure frequency reduction from baseline. Seizure freedom was defined as no seizures since at least the prior visit; therefore, seizure freedom rates at 3, 6 months, and the last visit represent the percentages of patients who had no seizures for ≥3 months, and the seizure freedom rate at 12 months represents the percentage of patients who had no seizures for ≥6 months. Statistical comparisons were conducted using the Chi-squared test. *AED* antiepileptic drug

The number of concomitant AEDs used at baseline was known for 2045 patients. Of these, the numbers of patients treated with zero, one, two, and at least three concomitant AEDs were 88 (4.3%), 969 (47.4%), 567 (27.7%), and 421 (20.6%), respectively (Table 2). Therefore, 1057 (51.7%) patients were treated with less than two concomitant AEDs and 988 (48.3%) were treated with at least two concomitant AEDs. At all timepoints, responder and seizure freedom rates were significantly greater in patients treated with less than two versus at least two concomitant AEDs (Fig. 6).

The overall incidence of AEs was significantly lower in patients treated with less than two versus at least two concomitant AEDs [30.7% (321/1047) vs. 38.0% (369/971); *χ* ^2^ = 12.07; *p* = 0.001; Chi-squared test]. In addition, the rate of ESL discontinuation was significantly lower in patients treated with less than two versus at least two concomitant AEDs [9.7% (98/1008) vs. 18.0% (169/941); *χ* ^2^ = 27.93; *p* < 0.001; Chi-squared test].

## Discussion

This retrospective pooled analysis of data from over 2000 European patients demonstrated that ESL was effective and generally well tolerated when used as an adjunctive treatment for partial-onset seizures under real-world clinical practice conditions over a median duration of >5 years. After 12 months of ESL treatment, 73.4% of patients were retained on ESL, 75.6% of patients had responded to treatment, and 41.3% of patients had been seizure free for ≥6 months. ESL treatment resulted in significant reductions from baseline to the last visit in the median frequencies of total, simple partial, complex partial, and secondarily generalized seizures (*p* < 0.01 for all). The proportions of patients with unchanged or worsened seizure frequencies remained low and relatively stable for the duration of the study (<15% and <11% of patients at all timepoints, respectively).

The most important finding of this study was that no unexpected safety signals emerged when ESL was used over the long term as adjunctive therapy for partial-onset seizures under real-world conditions. The safety profile of ESL was consistent with that observed in clinical trials [1, 32]. The most commonly reported AEs (≥5% of patients) were dizziness (6.7%), fatigue (5.4%), and somnolence (5.1%), and the AEs leading to ESL discontinuation in ≥2% of patients were dizziness (2.3%) and fatigue (2.0%). Real-world studies complement evidence from clinical trials by providing information on how an AED performs in clinical practice, where patients are more diverse than those recruited for clinical trials and treatment is individualized on a patient-by-patient basis, rather than being administered according to a pre-defined study protocol. In addition, they allow long-term surveillance monitoring for the potential emergence of idiosyncratic AEs that are rare and/or take time to develop. The importance of long-term post-marketing surveillance is highlighted by the findings of studies conducted for other AEDs. For example, a study with felbamate reported serious side effects of aplastic anemia and hepatic toxicity [33], and studies with retigabine (ezogabine) found idiosyncratic AEs of retinal pigment abnormalities and/or blue-grey discoloration of the lips, nail beds, hard palate, and conjunctiva emerged following long-term use [34, 35]. This study’s findings, therefore, provide evidence indicating that ESL’s safety profile is predictable and consistent with long-term use. It is also noteworthy that the size of the patient cohort included in this study exceeds the number of patients included in ESL clinical trials (approximately 1800 patients in total [1]). As such, the study provides robust evidence of ESL’s performance in the clinical practice setting.

Hyponatremia has been reported as a common AE in patients treated with ESL in clinical trials (1.2%) [1], and higher rates of hyponatremia have been reported in elderly patients (8.3%) [36]. In the current analysis, hyponatremia was reported as an AE in 3.5% of patients and led to discontinuation in 1.0% of patients. The slightly higher rate of hyponatremia reported in the clinical practice setting, in comparison with clinical trials, is perhaps unsurprising, given that the study population was more diverse in terms of age (11.7% ≥ 65 years) and comorbidities than that recruited for the randomized, controlled clinical trials. However, it is good practice to monitor for the potential development of hyponatremia with ESL treatment through laboratory testing, particularly in the elderly. Psychiatric disorders have uncommonly been reported as AEs in ESL clinical trials [1]. In the current analysis, 3.3% of patients reported psychiatric AEs. All AEDs may have effects on mood and behavior in patients with epilepsy [37], and, in comparison with some other AEDs, the observed incidence of psychiatric AEs in the current study was relatively low. For example, levetiracetam is commonly associated with psychiatric AEs including depression, aggression, anxiety, and irritability [38], and in a study of 517 adult patients treated with levetiracetam, 10.1% developed psychiatric AEs [39]. Of the patients who initiated ESL due to poor tolerability to previous AEDs, only a minority (12.0%) discontinued ESL due to AEs. The overall high retention rate observed in the study is a further indication that ESL was well tolerated in the clinical practice setting. Differences in the safety profiles of AEDs may be reflective of their particular structures and modes of action [40, 41].

An important aspect of this study was that it included the largest population of patients treated with ESL in clinical practice to have been investigated to date, allowing meaningful subgroup analyses to be conducted. When the data were analyzed in terms of age at study entry, the effectiveness of ESL treatment was found to be significantly superior in elderly patients (≥65 years) compared with non-elderly patients (<65 years). The favorable effectiveness of ESL in elderly patients is consistent with previous findings from a multicenter, open-label, non-controlled, single-arm, Phase III trial, in which the responder and seizure freedom rates during a 26-week maintenance period were 54.9 and 15.5%, respectively [36]. Studies of other AEDs have also demonstrated that epilepsy in elderly patients can be managed effectively, often using lower doses than those shown to be efficacious in younger patients [42, 43]. This may be because plasma concentrations of AEDs tend to be higher in older versus younger patients, due to age-related physiological changes in, for example, protein binding, intestinal transit time, and drug elimination [44–46]. Age-related changes also have an impact on AED tolerability; for example, hepatic and renal impairment affect the pharmacokinetic and pharmacodynamic properties of AEDs, increasing the likelihood of side effects, and high levels of comorbidity and polypharmacy increase the likelihood of drug–drug interactions and associated toxicity [44, 47]. It is, therefore, perhaps unsurprising that the incidence of AEs in the current study was significantly greater in patients aged ≥65 years compared with those aged <65 years. However, it is encouraging that the rate of ESL discontinuation due to AEs was not significantly higher in those aged ≥65 versus <65 years, indicating that the AEs experienced by elderly patients were generally tolerable and/or managed effectively. A potential advantage of ESL in the geriatric setting is that it is administered once daily, reducing medication burden and increasing the likelihood of treatment adherence, in comparison with agents requiring multiple dosing per day [48].

The subgroup analysis in which data were compared based on the number of concomitant AEDs that patients were receiving when ESL therapy was started (as a marker for treatment refractoriness) showed that, at all timepoints, responder and seizure freedom rates were significantly greater in patients treated with less than two versus at least two concomitant AEDs. These findings are consistent with those of the Eslicarbazepine acetate in Partial-Onset Seizures (EPOS) study (one of those included in the current analysis), which showed that ESL was effective as the only add-on to monotherapy in patients with refractory partial-onset seizures [21]. The incidence of AEs and the rate of ESL discontinuation due to AEs were significantly lower in patients treated with less than two versus at least two concomitant AEDs. These results are consistent with the recognition that AEs and pharmacokinetic interactions may become more frequent as patients’ drug burden is increased, supporting recommendations to reduce levels of polytherapy wherever possible [49, 50]. It is, therefore, encouraging that there was a significant reduction in the number of concomitant AEDs used from baseline to the last visit in the current study.

When the data in this study were analyzed in terms of the mode of action of patients’ concomitant AEDs, it was found that, at all timepoints, responder and seizure freedom rates were significantly greater in patients who were not receiving treatment with other SCBs, compared with those who were receiving treatment with other SCBs. This observation supports the idea of ‘rational polytherapy’, where AEDs with different modes of action may synergize in terms of effectiveness when used in combination [50, 51]. It is, however, noteworthy that ESL was also effective in patients treated with other SCBs, with responder and seizure freedom rates of 70.2 and 28.6% after 12 months, respectively. These findings may reflect differences in the effects of ESL on voltage-gated sodium channels in comparison with other SCBs, since ESL selectively enhances slow inactivation of sodium channels, whereas other SCBs, such as carbamazepine and oxcarbazepine, alter the fast inactivation of sodium channels [3]. Indeed, experimental models have demonstrated that ESL is able to overcome cellular resistance to carbamazepine [52], which is consistent with clinical findings demonstrating that ESL can be effective in patients who have previously not achieved sufficient seizure control with carbamazepine [53, 54].

This study was additionally able to provide insights into treatment practice when ESL is used in clinical practice. In the majority of patients, ESL was initiated due to the lack of effectiveness of previous AED therapy, although poor tolerance to previous treatment was also an important consideration for this choice of treatment. ESL was initiated at a median dose of 400 mg/day. Although the rate of titration was not analyzed, the median maintenance dose was 800 mg/day from month 3 onwards. These median initiation and maintenance doses are consistent with approved recommendations for the adjunctive treatment of adults with partial-onset seizures [1] (and almost 90% of patients were aged 18–64 years). However, 8.9 and 0.9% of patients received an ESL dose of >1200 and >1600 mg/day, respectively, at some point during the observation period, which is above the maximum dose recommended for use as adjunctive therapy (1200 mg/day) [1]. ESL is additionally approved as monotherapy for the treatment of partial-onset seizures, up to a maximum dose of 1600 mg/day [1, 2]. A minority of patients in the current study (4.3%) received ESL as initial monotherapy and 17.1% of patients were receiving ESL as monotherapy at the final visit. It is currently not known whether higher doses of ESL were used in patients treated with monotherapy in the present study, as analysis of data from this patient subgroup is currently ongoing.

As a retrospective pooled analysis, this study has acknowledged limitations. As outlined previously, there was great heterogeneity in the objectives of the studies included and in the information they reported. Moreover, individual patient data were not reviewed systematically post hoc (although the data were previously reviewed by the authors of the individual studies). Nevertheless, some of the limitations of pooled analysis are likely to have been mitigated by the large number of patients included in the study population, which allowed robust statistical methodology to be employed and meaningful treatment effects to be revealed, providing long-term, real-life information on a single AED. It should also be noted that seizures were classified according to the 1981 recommendations of the International League Against Epilepsy [30], rather than its more recent recommendations [55], since this was the classification system originally used by the study centers involved in the clinical practice studies that comprised the analysis.

In summary, in this study, which is the largest ESL clinical practice study conducted to date, no unexpected safety signals emerged when ESL was used as adjunctive therapy for partial-onset seizures over a median duration of follow-up of >5 years. The safety profile of ESL in the clinical practice setting was found to be consistent with findings from clinical trials. ESL was shown to be an effective treatment when used under real-world conditions, with >30% of patients achieving seizure freedom at the last visit. These findings provide strong and reassuring evidence that complements data from the clinical trials, supporting the use of ESL for the treatment of partial-onset seizures.
